# Crystal structure investigation of La_5.4_W_1−y_Mo_y_O_12−δ_ for gas separation by high-resolution transmission electron microscopy

**DOI:** 10.1038/s41598-019-39758-2

**Published:** 2019-03-01

**Authors:** K. Ran, W. Deibert, M. E. Ivanova, W. A. Meulenberg, J. Mayer

**Affiliations:** 1 Central Facility for Electron Microscopy GFE, RWTH Aachen University, 52074 Aachen, Germany; 2 Ernst Ruska-Centre for Microscopy and Spectroscopy with Electrons ER-C, Forschungszentrum Jülich GmbH, 52425 Jülich, Germany; 3 Institute of Energy and Climate Research IEK-1, Forschungszentrum Jülich GmbH, 52425 Jülich, Germany; 4 Faculty of Science and Technology, Inorganic Membranes, Universtiy of Twente, 7500 AE Enschede, The Netherlands

## Abstract

Lanthanum tungstate (LWO) and LWO with 20 at.% and 35 at.% molybdenum substituting tungsten were prepared by the Pechini method. Phase purity and successful Mo substitution inside these dense LWO membrane materials were confirmed by conventional and high resolution transmission electron microscopy techniques. The split of La_2_/W_2_ site by around 0.3 Å was proven. Extra reflections show up in the diffraction patterns from Mo-substituted LWO, and together with simulations, these reflections were recognized as forbidden reflections in a non-substituted LWO system, while the extinction rules are broken by Mo substitution due to the different scattering factors of W and Mo. Energy-dispersive X-ray chemical mapping allowed direct visualization of individual atomic columns, and revealed that all Mo is located at the W_1_ sites in the Mo-substituted LWO. Moreover, the diffuse scattering in diffraction patterns provides direct evidence of short range clustering of oxygen vacancies and could be further related to the oxygen conduction of the LWO membranes.

## Introduction

Mixed proton-electron conductors are promising gas separation membranes and have attracted great research interests^1–5^. Comparing with membranes based on precious metal alloys or polymers, ceramics-based membranes offer certain advantages, such as better thermal and mechanical stability, long-term durability, as well as cost effectiveness. So far, the most widely studied oxide-based ceramic materials are perovskites, where several acceptor-doped cerates^6–8^ and zirconates^9,10^ show good ambipolar conductivity and relatively high hydrogen flux. However, stability of the cerates in CO_2_, H_2_O and S-containing atmospheres and the low grain-boundary conductivity of zirconates always present a critical issue with respect to their application.

As an alternative candidate, much attention has been recently given to lanthanum tungstate (LWO)^11–13^. A general formula to describe LWO is La_28−x_W_4+x_O_54+δ_v_2−δ_, where x is the amount of tungsten sitting on a lanthanum site, which in turn determines how many oxygen vacancies (v) are in the structure as inherent defects^14,15^. The LWO holds appreciable mixed proton and electron conductivity, thermal and hydrothermal stability as well as chemical stability in reducing and water-containing atmospheres at elevated temperatures. It has been pointed out that single-phase LWO can be prepared at 1500 °C with a La/W ratio in the window between 5.3 and 5.7^16,17^. Outside this compositional range, segregation of either La_2_O_3_ or La_6_W_2_O_15_ was found. While the formation of La_2_O_3_ is accompanied by a volume expansion that destroys the membrane in a relatively short time, the presence of La_6_W_2_O_15_ leads to intensive crack formation and complete disintegration of the membrane^18^.

At low and intermediate temperatures, LWO is a relatively pure proton conductor caused by hydration of intrinsic oxygen vacancies. For its application as gas separation membrane at higher temperatures, where mixed conductivity is required, electronic conductivity becomes the main limiting factor. In order to optimize the LWO performance, enhancing its electronic conductivity while retaining similar proton conductivity would be desired. Among many studies, partial substitution of tungsten by a more reducible cation such as molybdenum or rhenium was reported to serve this purpose^11,15,17,19–21^. For example, the Mo-substituted LWO exhibits a predominantly n-type electronic and oxygen-ionic conductivity in the temperature range 500–800 °C, while its stability in reducing atmospheres was confirmed by various characterization methods^11,21^. Meanwhile, reducing the LWO membrane thickness is considered as an alternative way to improve its performance, and asymmetric membrane structures using porous MgO and LWO as substrates were prepared^22–24^.

In spite of extensive studies on the conducting properties of LWO or Mo/Re-substituted LWO and several reports determining their structural models by neutron and X-ray diffraction (XRD)^14,15,19,25,26^, only a few attempts were made to characterize the mixed conductors using transmission electron microscopy (TEM) with atomic resolution. At local scale, a certain degree of oxygen vacancy ordering was revealed by electron diffraction in La_5.7_WO_12−δ_ in contrast to La_5.3_WO_12−δ_, as lower tungsten content and therefore more oxygen vacancies in La_5.7_WO_12−δ_ is expected^14^. Phase purity and structural stability of La_5.5_WO_12−δ_ and La_5.5_W_0.8_Mo_0.2_O_12−δ_ were confirmed by TEM imaging before and after H_2_ permeation test^21^. Combining electron diffraction and high resolution TEM, nanodomains with an incipient superstructure were reported in La_28−y_(W_0.6_Mo_0.4_)_4+y_O_54+δ_, and when the Mo content goes higher, a new phase with rhombohedral structure was proposed^15,20^.

Here we performed a comprehensive TEM study to characterize the crystal structure of Mo-substituted LWO at atomic scale. The LWO membrane materials with nominal La/W ratio of 5.4 (in atomic percent) were prepared by the Pechini method^27^, where nominally 20 at.% and 35 at.% W was substituted by Mo respectively. Various techniques were employed, including high-angle annular dark-field (HAADF) imaging, energy-dispersive X-ray (EDX) chemical mapping, electron diffraction and simulations of HAADF images and diffraction patterns. The main focus is to precisely locate the Mo dopant and understand the LWO crystal structure before and after Mo substitution. The structural information revealed in this study has the potential to be further correlated with the gas separation performance of the Mo-substituted LWO membrane and could provide valuable information to achieve a better system with improved mixed conductivity, mechanical stability and tolerance for the harsh working environment.

## Results and Discussion

Three types of sintered pellets, pure LWO, LWO with nominally 20 at.% and 35 at.% of W replaced by Mo (LWO-Mo_00_, LWO-Mo_20_ and LWO-Mo_35_) were prepared and studied in this work. An even higher Mo content was found to degrade the membranes’ mechanical stability in preliminary experiments, and is therefore excluded from this study. The as-sintered pellets of the three types were first analyzed by XRD, in which all of them were proven to be phase pure within the experimental detection limit (Supplementary Fig. S1). Starting from the pellets, cross-section scanning electron microscope (SEM) samples were firstly prepared, which were used later for cutting lamellas by the focused ion beam (FIB) technique.

### Microstructure of non-substituted LWO, LWO-Mo_00_

**Figure 1:**
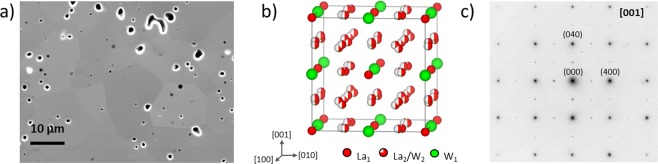
Structural information of LWO. (**a**) Cross-section SEM image of LWO-Mo_00_. (**b**) One unit cell of the average LWO model. Oxygen is not shown here. (**c**) Diffraction pattern from LWO-Mo_00_ along [001] zone axis, indexed by the model in (**b**).

Figure 1a shows a cross-section SEM image from the LWO-Mo_00_. Compact grains with irregular shapes and clean boundaries are visible. The grain size ranges from several micrometers up to tens of micrometers. Individual pores appearing as dark dots with mostly shiny edges, can be located both inside the grains and at the grain boundaries, without clear preference. The shiny edges are a result of charging, as carbon coating is less effective around the pores. Based on the SEM EDX measurement, no Mo was detected in LWO-Mo_00_ (Supplementary Figs S2 and S3). Thus, an average LWO structural model with a cubic fluorite-related structure^28^ belonging to the space group $Fm\bar{3}m$ (as will be discussed later), was employed to describe the LWO-Mo_00_ structure. As shown in Fig. 1b, W_1_ and La_1_ atoms form a face-centered cubic (fcc) lattice. The nearest W_1_ neighbors are linked by a La_2_ site, which is split into two sites and thus each one is only half occupied. An additional W atom (W_2_) sits on one of the La_2_ sites to stabilize the LWO phase. A diffraction pattern from a single LWO-Mo_00_ grain is shown in Fig. 1c, which can be fully indexed as recorded along [001] zone axis, based on the model shown in Fig. 1b.

### LWO-Mo_20_ and LWO-Mo_35_ by electron diffraction

Both the LWO-Mo_20_ and LWO-Mo_35_ show compact grains with similar sizes, clean grain boundaries and randomly distributed pores as LWO-Mo_00_ (Supplementary Fig. S2). SEM EDX results confirm the successful Mo substitution in both materials, and the content of Mo determined by electron probe microanalysis (EPMA) shows remarkable consistence with the nominal values (Supplementary Table S1). Moreover, the porosities among the three membrane materials are noticed to decrease as the Mo content increases (Supplementary Fig. S2).

**Figure 2:**
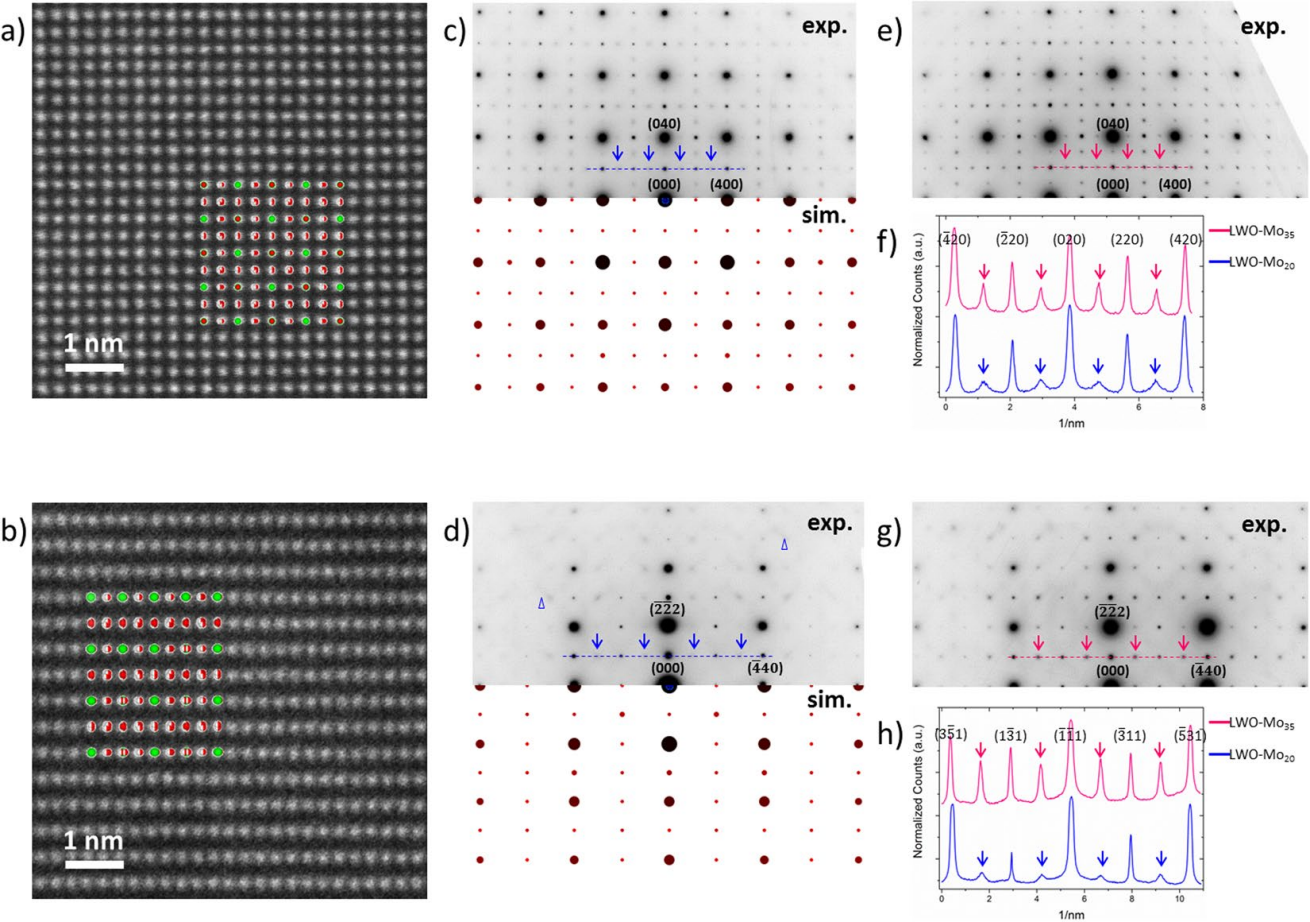
(**a**,**b**) HAADF images of LWO-Mo_20_ recorded along [001] and [112]. Insets are the average LWO model oriented correspondingly. (**c**,**d**) Comparison of the experimental pattern from LWO-Mo_20_ (upper part, exp.), and the simulated pattern (lower part, sim.) based on the average LWO model, along [001] and [112] zone axis respectively. (**e**) Diffraction pattern from LWO-Mo_35_ recorded along [001]. (**f**) Intensity profiles extracted from the dashed lines in (**c**) and (**e**). The blue/pink arrows mark the same reflections as the blue/pink arrows in (**c**)/(**e**). (**g**) Diffraction pattern from LWO-Mo_35_ recorded along [112]. (**h**) Intensity profiles extracted from the dashed lines in (**d)** and (**g**). The blue/pink arrows mark the same reflections as the blue/pink arrows in (**d**)/(**g**).

Figure 2a–d show HAADF images and diffraction patterns from LWO-Mo_20_, along [001] and [112] directions, respectively. The average LWO models are oriented correspondingly and placed as insets in Fig. 2a,b. Figure 2c,d compares the experimental (upper part, exp.) and simulated (lower part, sim.) diffraction patterns based on the average LWO model. While the HAADF images can still be qualitatively understood by the model, both experimental diffraction patterns show a distinct discrepancy from the simulations in Fig. 2c,d. In general, diffuse scattering is noticeable, as suggested by the blue triangles in Fig. 2d. In addition, not all the reflections can be indexed by the average LWO model, and some of them are marked by the blue arrows in Fig. 2c,d. For comparison, diffraction patterns were recorded from LWO-Mo_35_ along [001] and [112] as well, see Fig. 2e,g. Similar diffuse scattering and extra reflections show up in the LWO-Mo_35_ patterns. Along the dashed line in Fig. 2c,e, intensity profiles are extracted and plotted in Fig. 2f. For both LWO-Mo_20_ and LWO-Mo_35_, extra reflections arise at equal positions (midpoint between two indexed spots), and become apparently stronger in LWO-Mo_35_ than in LWO-Mo_20_. Intensity profiles are compared between the two [112] patterns as well in Fig. 2h. Again, extra reflections are noticed at corresponding positions, and show higher intensity in LWO-Mo_35_ than in LWO-Mo_20_. Such a behavior is consistently observed in the diffraction patterns from LWO-Mo_20_ and LWO-Mo_35_ along other zone axes as well (Supplementary Fig. S4).

### LWO-Mo_20_ and LWO-Mo_35_ by EDX chemical mapping

**Figure 3:**
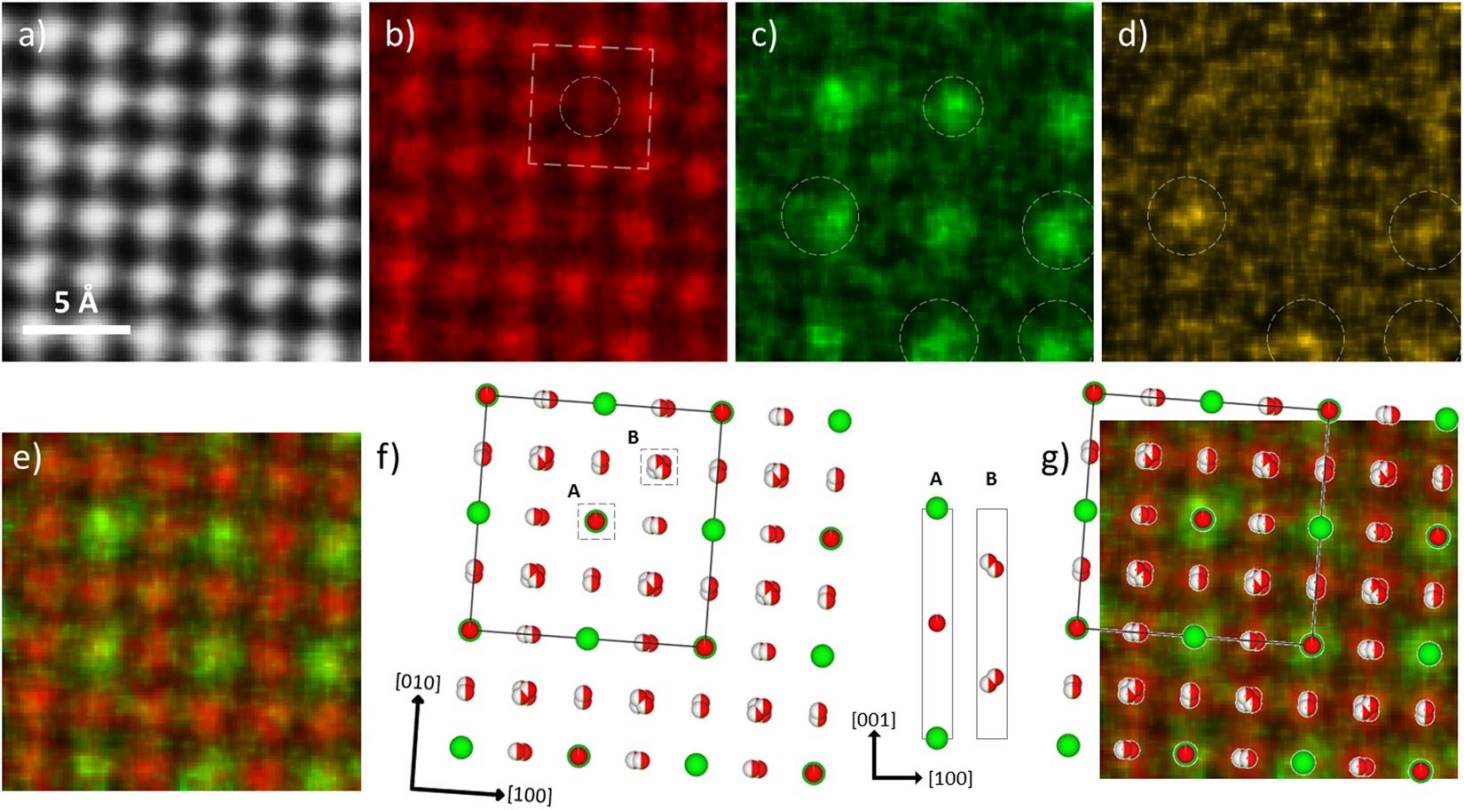
EDX chemical mapping of LWO-Mo_20_. (**a**–**d**) Simultaneously recorded HAADF image and maps from La *L* line, W *L* line and Mo *L* line from LWO-Mo_20_ along [001] zone axis. (**e**) Mixed La and W map. (**f**) The average LWO model oriented along [001], and the solid square defines one unit cell. Two types of atomic columns (A and B) are sketched on the right by vertically flipping the model. (**g**) Comparison between the average LWO model and the mixed La and W map.

Atomic resolution EDX chemical mapping was then carried out for all samples, and the results from LWO-Mo_20_ along the [001] zone axis are shown in Fig. 3. Figure 3a–d depict the simultaneously recorded HAADF image, and maps from the La *L* line, W *L* line and Mo *L* line, extracted with selected energy windows, respectively. Clearly, direct visualization of La and W atomic columns is possible. In Fig. 3b, the La is noticed to occupy each atomic column, but recognized with two different intensities. Each column with a relatively lower intensity and thus lower La content stays at the center of a square defined by eight other columns with higher intensity and thus higher La content, in Fig. 3b marked by the dashed circle and square. In contrast, the local maxima in the W map are all located at the less preferred La sites. The top circle in Fig. 3c is placed at the same position as the circle in Fig. 3b, and this coincidence is more evident in the mixed La and W map in Fig. 3e. The Mo map in Fig. 3d shows a quite low intensity due to its low concentration. However, the coincidence between local maxima of Mo and several W local maxima can still be determined unambiguously, as indicated by the circles which are placed at equal positions in Fig. 3c,d. Referring to the average LWO unit cell, there are two types of atomic columns (A and B) when viewed along [001] direction, as shown in Fig. 3f. The type A column contains one La_1_ and one W_1_ atom corresponding to the less preferred La sites (local maxima of W), and the type B consists of two split La_2_/W_2_ sites, corresponding to the more preferred La sites. The comparison in Fig. 3g between the average LWO model and the mixed La and W map, shows a good agreement, implying that the Mo substitution doesn’t change the LWO crystal structure.

**Figure 4:**
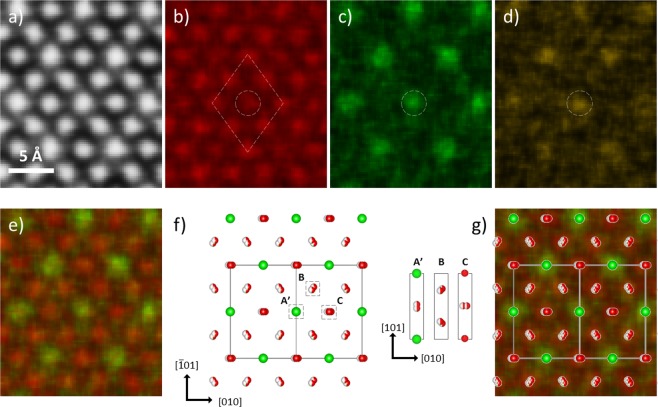
EDX chemical mapping of LWO-Mo_35_. (**a**–**d**) Simultaneously recorded HAADF image and maps from La *L* line, W *L* line and Mo *L* line from LWO-Mo_35_ along [101] zone axis. (**e**) Mixed La and W map. (**f**) The average LWO model oriented along [101], and the solid square defines one unit cell. Three types of atomic columns (A’, B and C) are sketched on the right by vertically flipping the model. (**g**) Comparison between the average LWO model and the mixed La and W map.

Along the [101] zone axis, EDX spectrum imaging was also applied to LWO-Mo_35_. Figure 4a–d show the simultaneously recorded HAADF image, and maps from the La *L* line, W *L* line and Mo *L* line. The La map in Fig. 4b shows again a La occupation of all atomic columns but with two types of intensities. The less preferred La site stays at the center of a rhombohedron defined by eight more preferred La sites, as indicated by the circle and rhombohedron in Fig. 4b. The less preferred La sites, local maxima of W and local maxima of Mo coincide with each other, and one example is indicated by the circles in Fig. 4b–d. Comparing with Fig. 3d, the local maxima of Mo in Fig. 4d show a clearly higher visibility and a more even coverage of the W_1_ sites. Three types of atomic columns are indicated in the average LWO model along [101] projection, as sketched in Fig. 4f. In addition to the aforementioned type B in Fig. 3f, two other types are expected. The type A’ column has a W_1_ and a split La_2_/W_2_. Similarly, the two atomic sites in type C columns are occupied by a La_1_ and a split La_2_/W_2_ respectively. However, the difference between type B and C is too small to be distinguished by EDX mapping, and therefore only two kinds of intensity are noticed in Fig. 4b. Again, the average LWO model and the mixed La and W map show a good agreement in Fig. 4g.

### Understanding the diffraction pattern

**Figure 5:**
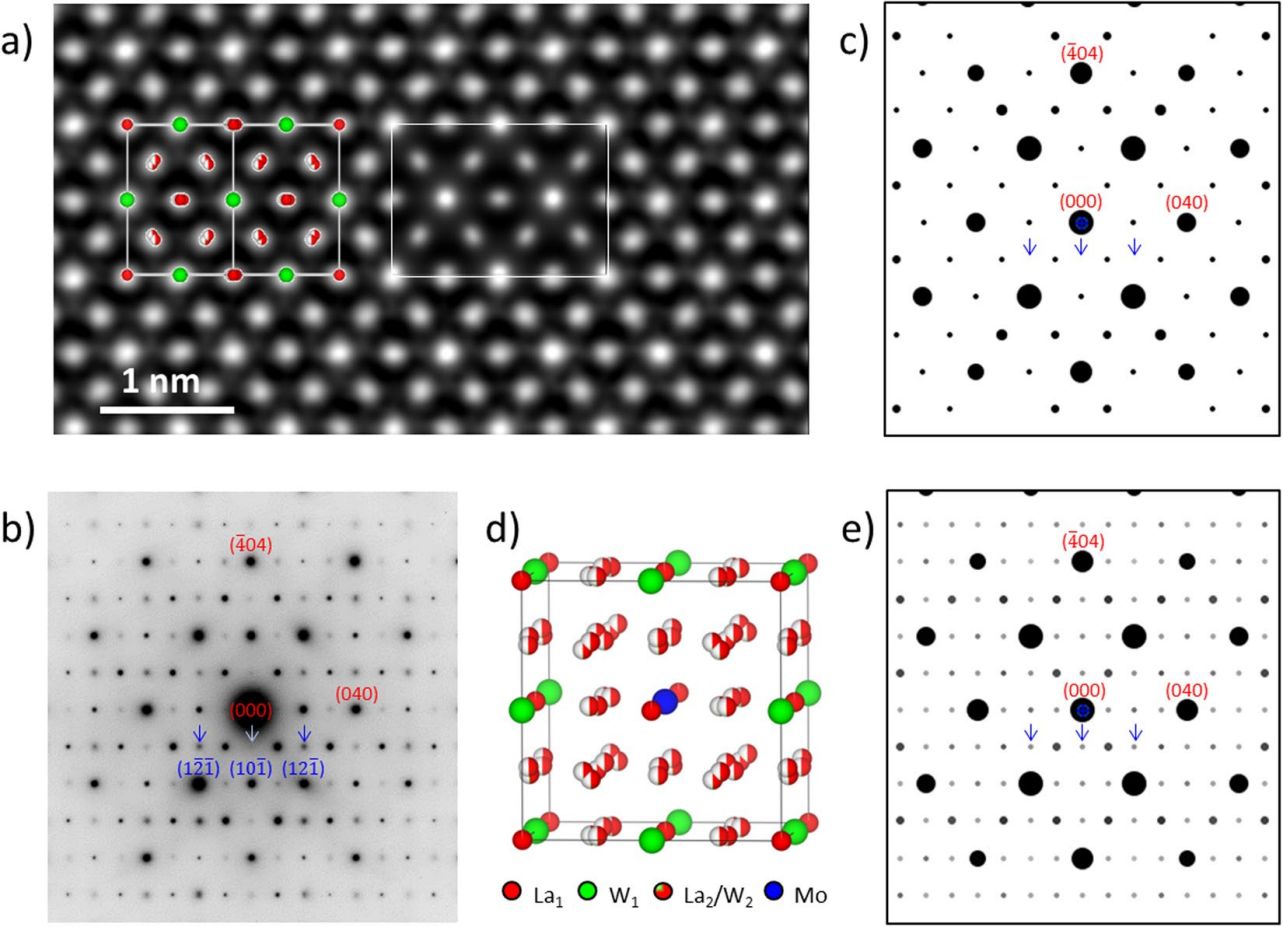
(**a**,**b**) HAADF image after averaging and smoothing, and diffraction pattern from LWO-Mo_35_ recorded along [101] zone axis. The insets in (**a**) are an average LWO model and a simulated HAADF image based on the model. (**c**) Diffraction pattern simulated based on the average LWO model along [101]. (**d**) The average LWO model with the center W replaced by a Mo atom. (**e**) Simulated diffraction pattern based on the substituted model in (**d**) along [101]. The arrows are placed at equal positions in (**b**), (**c**) and (**e**).

The HAADF images from both LWO-Mo_20_ and LWO-Mo_35_ show a good agreement with the average LWO model, yet provide limited information about the Mo distribution. As a complementary technique, EDX chemical mapping provides direct evidence on Mo replacing W, and thus suggests a substituted model for further study of the extra reflections in Fig. 2. Figure 5a,b are the HAADF image and diffraction pattern from LWO-Mo_35_ along the [101] zone axis. The image quality in Fig. 5a was improved through averaging by an iterative rigid alignment algorithm and smoothing by a nonlinear filtering algorithm^29^. A detailed process is described in the Supplementary Fig. S5. An average LWO model oriented correspondingly together with a simulated HAADF image based on the model are shown as insets in Fig. 5a. The diffraction pattern was simulated based on the same model, see Fig. 5c. Between the simulated and experimental image in Fig. 5a, no significant difference is noticed. However, when comparing the diffraction patterns in Fig. 5b,c, extra reflections show up again in the experimental pattern. Several extra reflections are marked by the arrows in Fig. 5b, and indexed based on their positions. Replacing the center W atom by a Mo atom, Fig. 5d suggests a feasible model for the Mo-substituted LWO, where the pristine LWO lattice is kept and a Mo/(W + Mo) ratio around 25 at.% is reached. Diffraction pattern was then simulated based on this substituted model along [101], see Fig. 5e. As marked by the arrows, similar extra reflections as in Fig. 5b exist in Fig. 5e.

To understand these extra reflections, the average LWO model can be further simplified as two sets of fcc lattices of La_1_ and W_1_, which are shifted by $\mathop{a}\limits^{\rightharpoonup }/2$ from each other. The La_2_/W_2_ sites barely show influence on the experimentally observed extra reflections, as similar diffraction patterns were simulated, based on models with and without La_2_/W_2_ sites (Supplementary Fig. S6). In addition, given the low occupancy of W at the La_2_/W_2_ sites, the chance that a Mo atom replaces a W atom at La_2_/W_2_ sites is even lower and thus can be ignored. This assumption is also supported by the chemical mapping in Figs 3 and 4, where local maxima of Mo were only located at the W_1_ sites. For LWO, the structure factor, *F*_*La*+*W*_, can be described as ${F}_{La+W}=\{{f}_{La}+{f}_{W}{e}^{\pi i(h+k+l)}\}\cdot {F}_{unit}$, where *f*_*La*_ and *f*_*W*_ are the atomic scattering amplitude for La and W atoms^30^ respectively, *h*, *k* and *l* are the Miller indices, and ${F}_{unit}=1+{e}^{\pi i(h+k)}+{e}^{\pi i(h+l)}+{e}^{\pi i(k+l)}$. *F*_*La*+*W*_ = 0, if *h*, *k* and *l* are mixed odd and even. Thus, the extra reflections in Fig. 5b indexed as ($1\bar{2}\bar{1}$), ($10\bar{1}$) and ($12\bar{1}$) should be all forbidden. Successful Mo substitution revised *F*_*La*+*W*_ into ${F}_{La+W+Mo}={f}_{La}\cdot {F}_{unit}+{f}_{W}{e}^{\pi i(h+k+l)}\cdot {F}_{unit}-{f}_{W}{e}^{\pi i(h+k+l)}+{f}_{Mo}{e}^{\pi i(h+k+l)}$, where *f*_*Mo*_ is the atomic scattering amplitude for Mo atoms. ${F}_{La+W+Mo}=\pm ({f}_{W}-{f}_{Mo}\,)$, if *h*, *k* and *l* are mixed. As a result, reflections such as ($\bar{1}\bar{2}1$), ($\bar{1}01$) and ($\bar{1}21$) with mixed Miller indices are allowed to appear, with an intensity proportional to ${({f}_{Mo}-{f}_{W})}^{2}$.

Comparing with LWO-Mo_20_, there will be more Mo in LWO-Mo_35_ contributing to the reflections with mixed Miller indices, which accounts for the generally stronger extra spots in the diffraction patterns from LWO-Mo_35_, in Fig. 2f,h. One other feature noticed in the experimental patterns is the diffuse scattering, which also becomes stronger with higher Mo substitution, as depicted in Fig. 2 and Fig. S4. Similar diffuse scattering has been reported in some other good oxygen-ion conductors with a cubic fluorite structure, such as ceria and zirconia^31,32^. In the lanthanide-doped ceria, the local ordering of oxygen vacancies develops with increasing doping level, and gives rise to stronger diffuse scattering. In addition, this development of ordering is found to be monotonously correlated with the degradation of ionic conductivity^20,33^. For LWO, a higher La/W ratio indicates more oxygen vacancies, but a less stable structure^14,34^. According to the EPMA results (Supplementary Table S1), LWO-Mo_35_ shows a higher La/(W+Mo) ratio, implying more concentrated oxygen vacancies than LWO-Mo_20_. Thus, within LWO-Mo_35_ the oxygen vacancies are more likely to cluster and get ordered, and therefore account for the stronger diffuse scattering observed experimentally.

### The average LWO model

Lanthanum tungstate was first believed to consist of La_6_WO_12_^35^ and only exists as a high temperature phase. Recent studies corrected this formula with a defected model, and mainly two (average) structural models were reported. Both of them describe the average crystal structure as an oxygen-deficient fluorite lattice, with doubling of the lattice parameter due to cation ordering. La_1_ and W_1_ form fcc lattices, and each La_1_ and W_1_ site is fully occupied and forms an octahedral environment with the surrounding oxygen atoms. The La_2_ has a more distorted environment with an average oxygen coordination of seven. The nearest W_1_ neighbors are linked by a La_2_ site, and one additional W_2_ sits on the La_2_ site to stabilize the LWO phase. Depending on whether the La_2_/W_2_ sites are split and thus half occupied, one model belongs to the space group $Fm\bar{3}m$^25^ and the other belongs to $F\bar{4}3m\,$^14,16,36^. The split of La_2_/W_2_ sites is considered to be necessary to develop a structural model on a local level^25^. Experimentally, studies based on synchrotron X-ray powder diffraction and neutron powder diffraction support the split model ($Fm\bar{3}m$) as a more accurate one than the non-split model ($F\bar{4}3m$)^19^. However, there is still no direct evidence thus far from TEM, to prove the split of La_2_/W_2_ sites.

**Figure 6:**
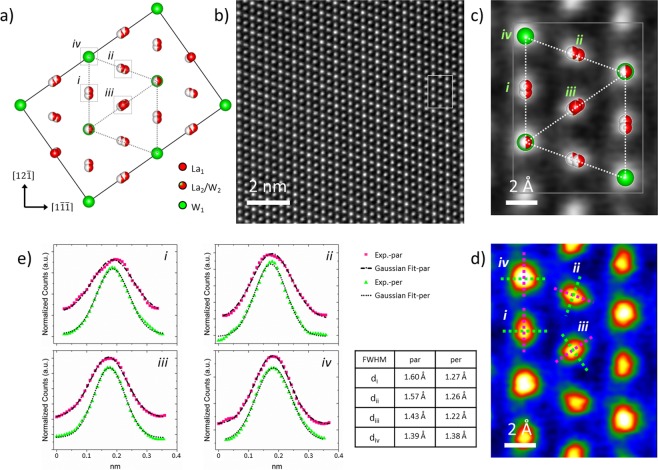
Measurement of the split of La_2_/W_2_ sites. (**a**) The average LWO model with split La_2_/W_2_ sites, oriented along [101] zone axis. Four different types of atomic columns are labeled as *i*, *ii*, *iii* and *iv*. (**b**) HAADF image from LWO-Mo_20_ recorded along [101] zone axis, after averaging and smoothing. (**c**) HAADF image cropped from the marked region in (**b**). The dotted rhombohedron in (**a**) is overlaid on the image for comparison. (**d**) The colored coded image of (**c**). The dotted pink/green lines are parallel/perpendicular to the edges and diagonal of the rhombohedron. (**e**) Intensity profiles extracted along the dotted lines in (**d**) for atomic columns *i* to *iv*, together with Gaussian fit results. The lower right table lists the full width at half maxima (FWHM) determined from Gaussian fit, for each atomic column along each direction.

Oriented along [101] zone axis, Fig. 6a shows the split model with its $[12\bar{1}]$ direction pointing upwards. A rhombohedron and one of its diagonals are defined by the dotted lines there, and four atomic columns are labeled as *i*, *ii*, *iii* and *iv*. Resulting from the split of the La_2_/W_2_ site, atomic columns elongate either along the edges or the diagonals of the rhombohedron, like those labeled as *i*, *ii* and *iii*. For the columns like the one labeled as *iv*, the elongation is along the [101] direction, and thus invisible under this condition. The HAADF image in Fig. 6b was recorded from LWO-Mo_20_ along the [101] zone axis. An ~1.07 × 1.38 *nm*^2^ area, as marked by the square, is cropped out and compared with the rhombohedron in Fig. 6c. The same image is then color coded in Fig. 6d, where the shape of atomic columns is better revealed. Already, the asymmetric feature in column *i*, *ii* and *iii* can be noticed. Intensity profiles along the dotted pink and green lines were extracted and plotted in Fig. 6e for each atomic column. The pink/green lines are parallel/perpendicular to the edges and diagonal of the rhombohedron. A Gaussian fit was then carried out, to estimate the full width at half maxima (FWHM) for each curve in Fig. 6e. The estimated values from each atomic column along both directions are then compared in the lower right table. Taking column *i* for example, the difference between d_*i*,par_ and d_*i*,per_ is around 0.33 Å, while for column *iv*, the d_*iv*,par_ and d_*iv*,per_ are almost the same (difference ~0.01 Å). Similarly, for column *i* and *iii*, around 0.3 Å difference between the parallel and perpendicular directions were determined. Thus, by HAADF imaging, the asymmetric elongation of atomic columns resulting from the La_2_/W_2_ site split is directly proven for the first time, which provides solid evidence for the accuracy of the split model.

## Conclusions

In summary, our study has revealed the atomic structure of LWO with different amounts of Mo substitution. The average LWO model with split La_2_/W_2_ sites was proven to be more accurate to describe the crystal structure. EDX chemical mapping confirmed that Mo replaces W, and up to 35 at.% W replaced by Mo didn’t affect the original fluorite structure of LWO. The different scattering factors of Mo and W resulted in extra reflections in the diffraction patterns from Mo-substituted LWOs. The diffuse scattering provided evidence of the clustering and short-range ordering of oxygen vacancies, which became stronger with higher Mo concentration.

Mo was introduced into LWO, aiming to improve the electrical conductivity of the membrane. However, this is accompanied with a side effect of ordering of oxygen vacancies at room temperature, which could degrade the membranes’ ionic conductivity. To enhance the electrical conductivity while maintain an acceptable ionic conductivity as well as the stability of the system, a comprehensive study on the membrane performance, the Mo substitution and the LWO crystal structure after substitution would be necessary. The atomic structures identified in this work can be further connected with the gas separation performance, and provide a solid structural basis for thoroughly understanding the membranes’ properties. Moreover, the Mo substitution shows the ability to alter the La/(W + Mo) ratio in a predictable way within a certain window, and additionally reduce the porosity of the membrane material. All these may in turn offer opportunities to rationally tailor the functional properties of the membranes by precise control of Mo content.

## Methods

### Preparation of LWO with and without Mo substitution

The LWO material was prepared by a modified Pechini method^27^. Lanthanum nitrate hexahydrate (Sigma Aldrich), ammonium tungstate (Sigma Aldrich) and ammonium molybdate (Sigma Aldrich) were used as the precursors. The complexation reaction was performed as shown in^11^. The mixtures were calcined at 900 °C in air to remove the organic constituents and form an oxide. Afterwards, 1 gram of the powder was pressed to pellets with a diameter of 20 mm. The final sintering step was performed at 1500 °C for 12 h with heating rates of 5 K/min to obtain high densification and complete phase formation. XRD measurements were performed with a Bruker D4 X-ray diffractometer. The Mo concentration was varied between 0 and 35%, meaning that up to 35% of the W was substituted by Mo. Higher Mo concentrations appeared to be not stable after storage in air for several days.

### TEM sample preparation

LWO pellets were first embedded in resin (Kulzer). After gradual grinding and polishing, cross section SEM samples with flat surfaces were prepared. Carbon coating was applied to reduce the possible charging problem during SEM imaging and EPMA measurement. TEM specimens were cut from the cross section SEM samples by focused ion beam (FIB) milling using an FEI Helios NanoLab 400 S system with a Ga ion beam^37^. Further thinning and cleaning were performed with an Ar ion beam in a Fischione Nanomill 1040 at 900 and 500 eV beam energy respectively.

### Characterization techniques

SEM and EPMA measurements were carried out on a JEOL JSM7000F and a CAMEBAX SX 50 instrument, respectively. Electron diffraction was performed on a FEI Tecnai F20 at 200 kV. High resolution HAADF imaging was conducted with an FEI Titan 80–300 STEM^38^ and an FEI Titan G2 80–200 ChemiSTEM^39^ microscope operated at 300 kV and 200 kV, respectively. Atomic resolution EDX chemical mapping was performed on an FEI Titan G2 80-200 ChemiSTEM microscope equipped with an XFEG, a probe C_s_ corrector and a super-X EDXS system. The convergence semi-angle for STEM imaging and EDX chemical mapping was approximately 22 mrad, while the collection semi-angle was 88-200 mrad for HAADF imaging. EDX maps were collected typically for ~10 minutes, and background subtraction was performed following the steps previously described^40^. STEM HAADF image simulations were performed with the Dr. Probe software package^41^. Aberrations were not taken into account in the simulations since this work was not aimed on full quantifying the experimental images. Structural models were visualized with VESTA^42^. Electron diffraction patterns were simulated by the JEMS software.

## Supplementary information

Supplementary Information
