# Supplementary material for: Crystal structure investigation of La5.4W1−yMoyO12−δ for gas separation by high-resolution transmission electron microscopy
Source: Sci Rep. 2019 Mar 1;9:3274. doi: 10.1038/s41598-019-39758-2 (PMC6397252; doi:10.1038/s41598-019-39758-2)
Supplement: Supplementary file 1 — Supplementary Information [file 41598_2019_39758_MOESM1_ESM.pdf]

Supplementary Information

Crystal structure investigation of $La_{5.4}W_{1-y}Mo_yO_{12-\delta}$ for gas separation by high-resolution transmission electron microscopy

K.Ran,[1,2*] W. Deibert,[3] M. E. Ivanova,[3] W. A. Meulenberg,[3,4] J. Mayer[1,2]

1. Central Facility for Electron Microscopy GFE, RWTH Aachen University, 52074 Aachen, Germany.

2. Ernst Ruska-Centre for Microscopy and Spectroscopy with Electrons ER-C, Forschungszentrum Jülich GmbH, 52425 Jülich, Germany

3. Institute of Energy and Climate Research IEK-1, Forschungszentrum Jülich GmbH, 52425 Jülich, Germany.

4. Faculty of Science and Technology, Inorganic Membranes, Universtiy of Twente, 7500 AE Enschede, The Netherlands.

*Correspondence and requests for materials should be addressed K.R. (email: ran@gfe.rwth-aachen.de)

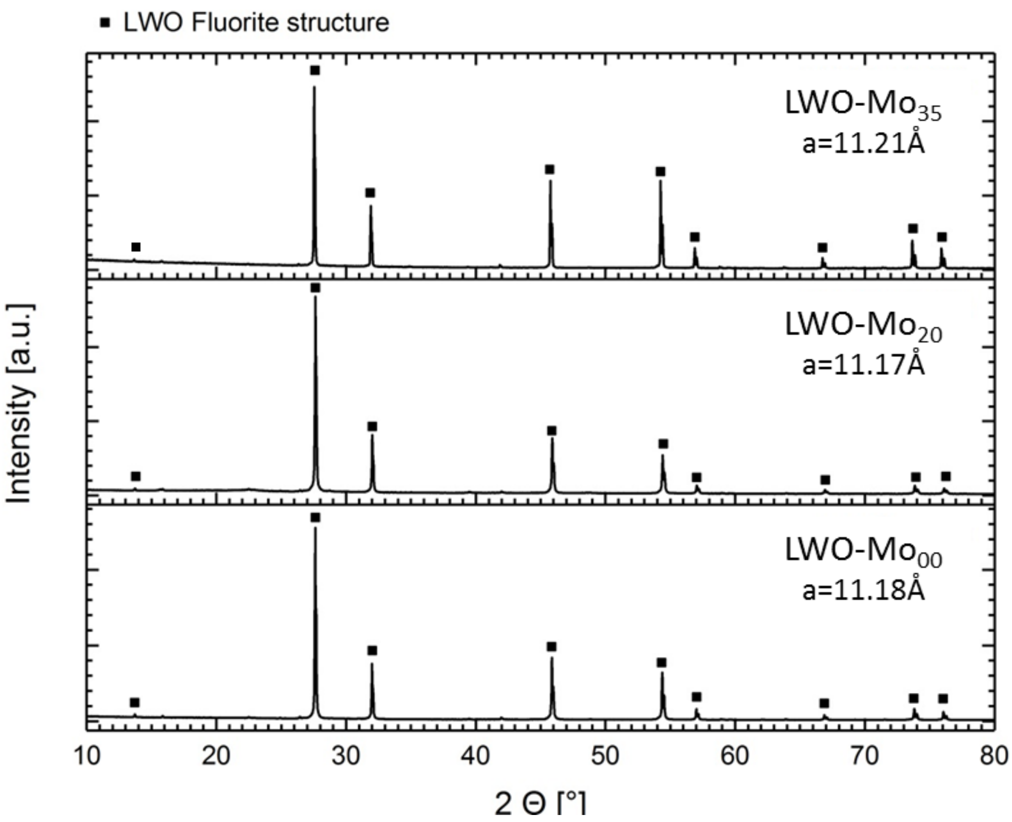

Figure S1: XRD results of the investigated material compositions LWO-Mo_{00}, LWO-Mo_{20} and LWO-Mo_{35}. Characteristic peaks for the LWO fluorite structure are marked.

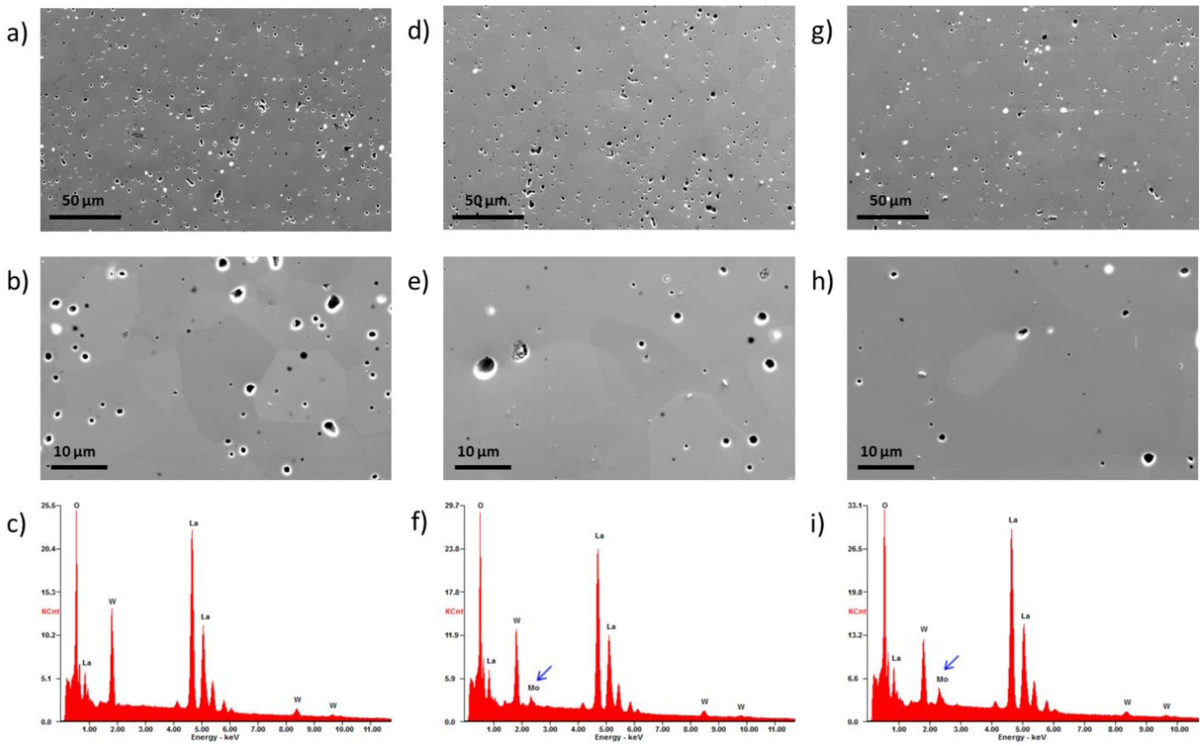

Figure S2. Cross-section SEM images and EDX measurement of LWO-Mo_{00} (a-c), LWO-Mo_{20} (d-f) and LWO-Mo_{35} (g-i). Arrows in (f) and (i) indicate the Mo peaks.

	LWO-Mo_{20}		LWO-Mo_{35}	
	EPMA	nom	EPMA	nom
La/(W+Mo)	5.55±0.05	5.4	5.93±0.05	5.4
Mo/(W+Mo)	0.21±0.01	0.2	0.36±0.01	0.35

TableS1. Microanalysis results given by the EPMA technique and compared to the nominal ratios, given in atomic percent.

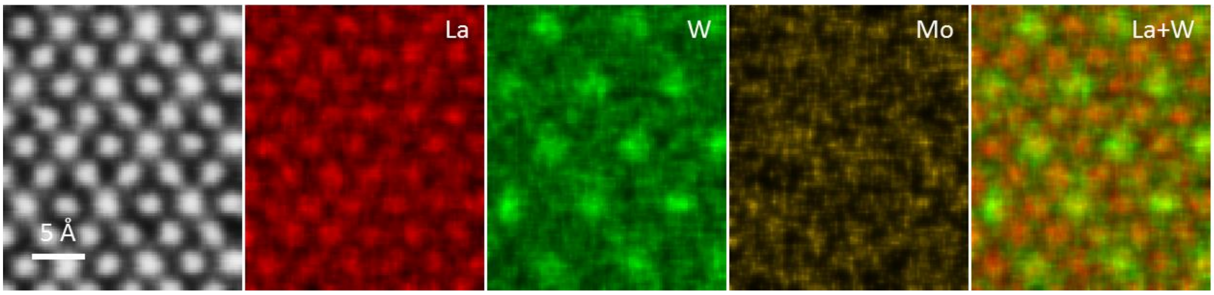

Figure S3. EDX mapping results from LWO-Mo_{00} along [101] zone axis. Left to right: the simultaneously recorded HAADF image and maps from La *L* line, W *L* line and Mo *L* line, together with the mixed La and W map. The La and W map are consistent with the average LWO model, while no significant signal can be detected in the Mo map.

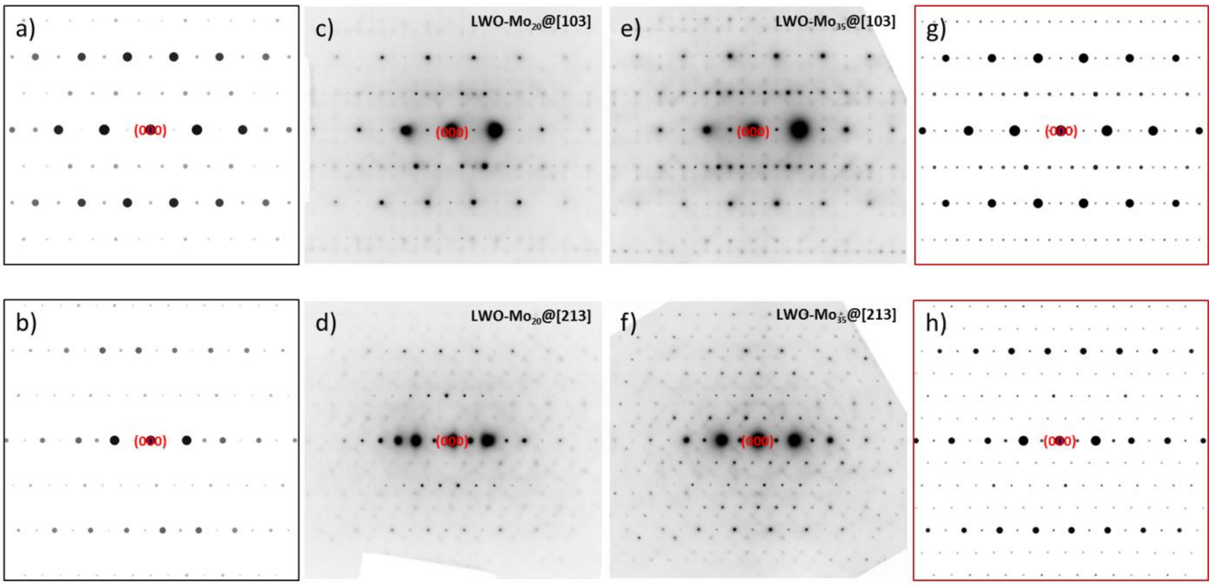

Figure S4. (a-b) Simulated diffraction patterns based on the average LWO model along [103] and [213] respectively. (c-d) Experimental patterns from LWO-Mo_{20} along [103] and [213]. (e-f) Experimental patterns from LWO-Mo_{35} along [103] and [213]. (g-h) Simulated patterns based on the model in Fig. 5d along [103] and [213].

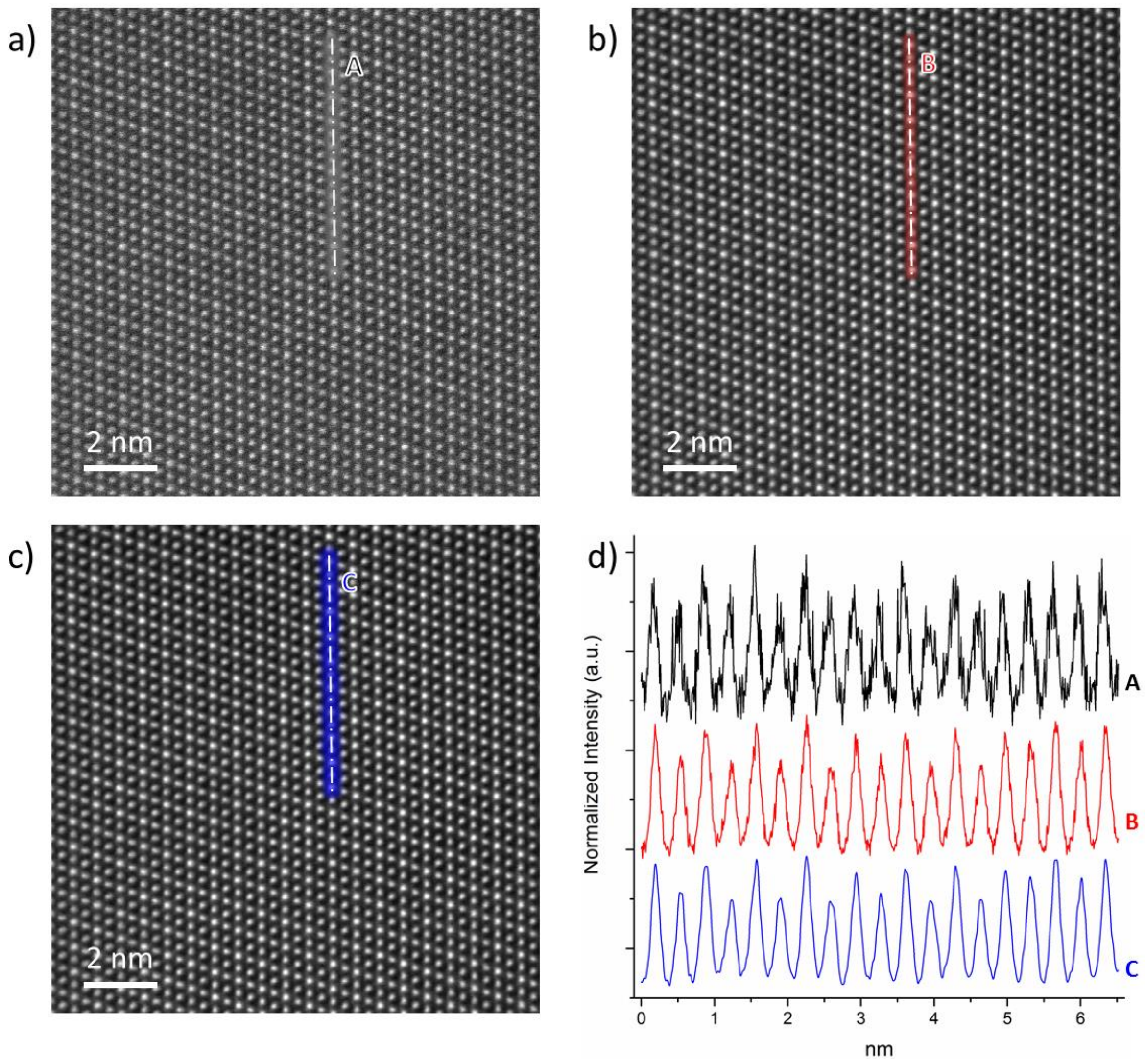

Figure S5. The image processing procedure. A set of ten HAADF images was recorded from LWO-Mo_{20} along its [101] zone axis with relatively short exposure time (2 s/frame). (a) One of the ten images. (b) Averaged image from the image set by an iterative rigid alignment algorithm. (c) The image in (b) is smoothed by a nonlinear filtering algorithm. (d) Extracted intensity profiles from the equal position in (a), (b) and (c).

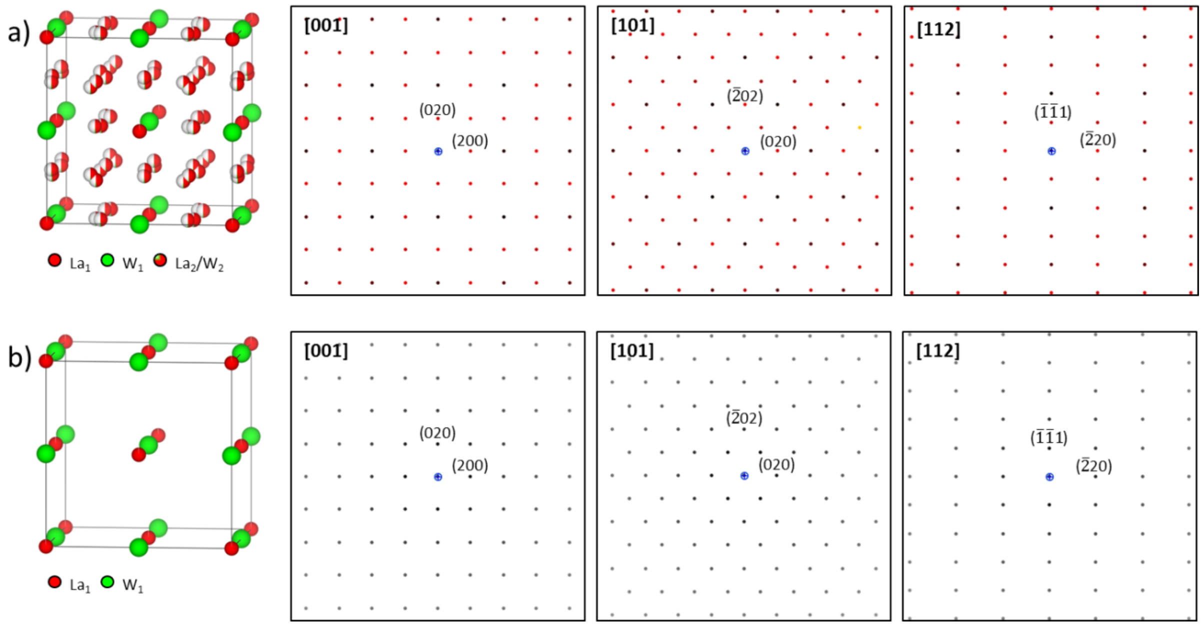

Figure S6. (a) One unit cell of the average LWO model and the simulated diffraction patterns along [001], [101] and [112] zone axis. (b) One simplified unit cell without the La_2/W_2 sites, and the simulated diffraction patterns along [001], [101] and [112] zone axis.
